# Surgical teams’ attitudes and opinions towards the safety of surgical procedures in public hospitals in the Brazilian Federal District

**DOI:** 10.1186/s13104-016-2078-3

**Published:** 2016-05-17

**Authors:** Heiko Thereza Santana, Maria Cristina Soares Rodrigues, Maria do Socorro Nantua Evangelista

**Affiliations:** National Health Surveillance Agency, SIA trecho 5, área especial 57, Brasilia, DF 71205-050 Brazil; Department of Nursing, Faculty of Health Sciences of the University of Brasilia (UnB), Campus Darcy Ribeiro, Brasilia, DF 70910-900 Brazil; Collective Health Department of the University of Brasilia (UnB), Brasilia, DF Brazil

**Keywords:** Patient safety, Adverse event, Surgery, WHO surgical safety checklist, Attitudes, Operating room, Health surveillance

## Abstract

**Background:**

According to the World Health Organization, the WHO surgical safety checklist can prevent complications, improve communication and contribute to postsurgical safety culture; hence, there is a need to investigate the attitudes and opinions of surgical teams regarding safety utilizing the WHO instrument. The aim of this study was to assess the attitudes and opinions towards surgical safety among operating room professionals in three public hospitals in the Brazilian Federal District.

**Methods:**

A cross-sectional study was conducted with the use of a checklist based on the safety attitudes questionnaire-operating room, sent out during the pre- and post-intervention surveys of the WHO surgical safety checklist (period I and period II) between 2012 and 2014.

**Results:**

About 470 professionals, mostly nurse technicians, responded to the questionnaire in both periods. Regarding the perception of safety and agreement about the collaboration of the operating team, a significant statistical improvement of the nursing staff and anesthesiologists was observed in the operating room after the checklist was implemented. After utilizing the checklist before each surgical procedure, concerns about patient safety and compliance with standards as well as rules and hand-washing practices in the operating room statistically improved after the post-intervention, especially by the nursing staff. The checklist was considered easy and quick to use by most respondents. They also believed that the checklist inclusion improved communication, reflecting significant differences. At least 90.0 % of respondents from each team agreed that the checklist helps prevent errors in the operating room.

**Conclusions:**

The study results showed progress in relation to the attitudes and opinions regarding surgical safety from operating teams in relation to the checklist response in the surveyed units. However, difficulties in its implementation are experienced, especially in relation to checklist use acceptance by the surgeons. New studies are needed to verify the sustainability of the surgical teams’ changes in attitudes in the hospitals studied.

## Background

Safety errors can cause damage and injuries to surgical patients, compromising their health during surgery, and can even lead to death [1, 2]. In the United States, 9000 sentinel events or “never events” were reported between 1990 and 2010, including: foreign objects left in a patient after a surgical procedure, wrong patient and wrong procedure, resulting in 6.0 % deaths, 32.9 % permanent and temporary (59.2 %) sequelae at a cost of 1.3 billion dollars [3, 4].

The WHO safe surgery saves lives program addresses the prevention of adverse events (AEs), which includes surgical site infection (SSI) prevention, safe surgical teams, safe anesthesia and surgical services [5]. This initiative, besides the administrative and managerial aspects of health care, prioritizes safety attitudes of surgical teams, given the complexities of the operating room (OR). Furthermore, patient safety culture indicates communication as its main tool, as established by the WHO surgical safety checklist, which can be applied not only to prevent surgical complications, but also to improve communication in the OR [6, 7].

In Brazil, a prospective transversal study (pre- and post-intervention) was conducted with the aim of evaluating the application of the WHO surgical checklist, involving 1141 (pre-intervention) and 1052 (post-intervention) patients, totaling 2193 patients [8]. Despite the findings of checklist item compliance variation in the surveyed hospitals, the implementation of the checklist as an intervention tool in the study showed good compliance for most items (such as identifying the patient, pulse oximeter placement, and pulse oximeter functioning had compliance levels above 95 %; introducing team members, surgeon hand antisepsis, and essential imaging display showed compliance levels above 92 %; procedure register and sample identification were above 76 %). Complications and deaths were low in both periods [8]. These findings may have contributed to the prevention of safety incidents and minimize risks during the implementation of the checklist in these services.

Given the sensitive nature of surgical care, surgical procedures require integration, communication and multidisciplinary team work among surgeons, anesthesiologists and nursing staff. Therefore, the WHO recommends the use of a surgical safety checklist to improve patient safety [5]. Haugen et al. [9] noted that the attitudes and behaviors of surgical teams reflect the development of safety procedures in the OR. The question to be asked is the following: to what extent do the surgical teams’ attitudes regarding safety contribute to the improvement or to the reduction of safety in the OR?

Studies relating team work to cohesion of the surgical team and to safety culture reveal a reduction in patient morbidity and mortality [10]. Also, assessments of attitudes and opinions of surgical teams about quality care and patient safety in the OR identified communication gaps between professionals [6, 11–15], which provide opportunities for changes in surgical procedures, excluding the empowerment of surgeons over the other members of the team and reducing gender conflict, which should improve levels of responsibility for complying with items on the list.

Conducting a survey on safety attitudes and morbidity and mortality reduction with the safety attitudes questionnaire-operating room (SAQ-OR) [16], Haynes et al. [6] found high levels of good attitudes in the OR.

An initiative of the Brazilian Health Surveillance Agency (ANVISA) involved, at first, the implementation of the WHO surgical safety checklist in public teaching hospitals in the Brazilian Federal District (DF) as a pilot project to promote strategies for the expansion of this initiative to other health services in the country. This study aims to evaluate the attitudes and opinions regarding surgical safety among operating room professionals in these hospitals before and after implementation of the checklist.

## Methods

### Research design

A cross-sectional study was performed in surgical centers in three public hospitals in the Federal District Department of Health, in the Central-West region of Brazil, between 2012 and 2014.

The hospitals for the study were chosen based on the following criteria: be public; belong to ANVISA’s sentinel network; and have one or more ORs. Hospital one is a district teaching hospital with a high level of care and has 748 beds. The health unit has 16 ORs and performs 7267 surgeries/year in the following areas: proctology, urology, orthopedics, vascular surgery, plastic surgery, gynecology, in addition to neurological surgery, cancer, trauma, and organ transplants. Hospital two is a federal teaching unit, regarded as a general hospital, which cares for medium to high-risk patients and 299 beds. It has ten ORs and performs 2905 surgeries/year in the following areas: general surgery, head and neck surgery, proctology, urology, orthopedics, vascular surgery, plastic surgery and gynecology. Hospital three is a district teaching unit with 226 beds. It has five ORs and performs 3695 surgeries/year in general surgery, proctology, urology, orthopedics, among others.

### Participants

The study population consisted of 472 health professionals working in three surgical centers (surgeons, anesthesiologists, surgical technologists, nurses, nursing technicians and nursing assistants, resident physicians, medical and nursing students, as well as heads of medical and nursing services) from the hospitals surveyed. Professionals from the surgery team included the chief physician or chief nurse, undergraduate or graduate students who would be scheduled to work for at least 2 weeks in the operating room or during the data collection period. Exclusion criteria included professionals who were absent due to vacations or other absences during the data collection. Professionals were interviewed in their workplaces when they were available for participation in the study. The interviews were conducted by trained professional nurses.

### Data collection

Data were collected through an attitude and opinion assessment tool about surgical safety in health services, based on the SAQ-OR modified questionnaire, including items regarding patient safety perception, communication and teamwork, as well as some questions about checklist implementation [6]. It is important to highlight that in 2011 a content validation study for Brazilian public hospitals under a cross-cultural perspective (SAQ-short form) showed moderate to strong correlation in each domain for all of the variables, except for the item “stress recognition” [17].

The instrument was concurrently applied while a prospective cross-sectional study was conducted with pre- and post-intervention (period I—pre-intervention and period II—post-intervention) of the WHO surgical safety checklist, based on ANVISA’s project safe surgery saves lives. All of the respondents are aware of the surgical safety checklist and had been previously trained on its use. After the conclusion of the pre-test stage, the questionnaire was applied 2 weeks before the beginning of period I and 2 weeks after the end of period II.

### Data analysis

The p value was calculated using the Mann–Whitney test for the differences in the distribution of periods I and II. Patients used an online survey builder (survey monkey) to enter their data, which was revised by the researcher. The database was set up with SPSS software, version 11.5 for Windows.

## Results

Considering all of the surgical teams working in the hospitals in this study, the response rate was 82 % in period I and 75 % in period II.

Out of 472 participants from surgical teams and heads of services, 257 were interviewed before intervention (period I) and 215 post-intervention (period II). The gender distribution was predominantly female in both periods, with no statistical difference, except for hospital 1 (Table 1).

**Table 1:** Socio-demographic characteristics of surgery teams according to period of intervention using the WHO Surgical Safety Checklist

Socio-demographic characteristics	Hospital 1				Hospital 2				Hospital 3				Total			
	Period I		Period II		Period I		Period II		Period I		Period II		Period I		Period II	
	No.	%	No.	%	No.	%	No.	%	No.	%	No.	%	No.	%	No.	%
Sex^a^																
Male	38	36.2	36	36.7	42	56.8	35	50.7	41	52.6	20	47.6	121	47.1	91	43.5
Female	67	63.8	62	63.3	32	43.2	34	49.3	37	47.4	22	52.4	136	52.9	118	56.5
Total	105	100.0	98	100.0	74	100.0	69	100.0	78	100.0	42	100.0	257	100.0	209	100.0
p value^c^	0.936				0.471				0.607				0.446			
Age^b^																
Mean	38.2		40.5		37.6		36.7		35.5		34.8		37.2		38.4	
Median	35.0		38.0		35.0		31.0		32.0		32.0		35.0		36.0	
Standard deviation	9.8		9.7		10.9		12.1		10.4		9.0		10.3		10.5	
Minimum	24.0		26.0		22.0		22.0		22.0		25.0		22.0		22.0	
Maximum	77.0		79.0		63.0		67.0		65.0		57.0		77.0		79.0	
IQR (Q3–Q1)	14.0		14.0		18.0		19.5		15.0		13.5		15.8		16.0	
Total	104.0		113.0		55.0		61.0		77.0		41.0		236.0		215.0	
p value^c^	0.035				0.361				0.872				0.223			
Occupation																
Anesthesiologist	7	6.7	6	5.3	14	15.7	10	13.2	14	17.9	5	11.9	35	12.9	21	9.1
Nurse assistant	14	13.3	16	14.0	6	6.7	4	5.3	3	3.8	4	9.5	23	8.5	24	10.3
Surgeon	7	6.7	8	7.0	27	30.3	8	10.5	12	15.4	5	11.9	46	16.9	21	9.1
Nurse	9	8.6	8	7.0	2	2.2	1	1.3	1	1.3	1	2.4	12	4.4	10	4.3
Instrument nurse	11	10.5	10	8.8	6	6.7	3	3.9	4	5.1	1	2.4	21	7.7	14	6.0
Other (specify)	4	3.8	1	0.9	9	10.1	11	14.5	15	19.2	5	11.9	28	10.3	17	7.3
Resident anesthesiologist	7	6.7	11	9.6	3	3.4	0	0.0	6	7.7	7	16.7	16	5.9	18	7.8
Resident surgeon	14	13.3	14	12.3	9	10.1	19	25.0	11	14.1	7	16.7	34	12.5	40	17.2
Resident nurse	1	1.0	0	0.0	0	0.0	0	0.0	0	0.0	0	0.0	1	0.4	0	0.0
Nurse technician	31	29.5	40	35.1	13	14.6	20	26.3	12	15.4	7	16.7	56	20.6	67	28.9
p value^c^	0.888				0.007				0.631				0.055			

*IQR* interquartile deviation

^a^Without response 7.5 % of the respondents

^b^Without response 10.5 % of the respondents

^c^p value was calculated based on the Mann–Whitney test for the distribution difference in periods I and II

The average age of respondents in the pre-intervention period was 37.2 and 38.4 years in the post-intervention period, without statistical significance. In relation to hospitals, most of them showed no significant difference between the mean ages and the periods, except for hospital 1, where, for period I, the mean was 38.2 years and for period II, 40.5 years (Table 1). The interquartile range was from 15.8 to 16.0, considering the 1st and 2nd quartiles. Work experience among interviewed healthcare professionals did not differ statistically between the two periods; the average for period I was 12.4 years and for period II, 11.5 years. In general, regarding work experience, there was no difference between the average years of experience in the current unit for the assessed periods. The average for period I was 7 years and for period II, 6 years (Table 1).

Most respondents from period I were nursing technicians (20.6 %), followed by surgeons (16.9 %), anesthesiologists (12.9 %) and surgery resident physicians (12.5 %). In period II, the nursing technician category was predominant (28.9 %), followed by surgery resident physicians (17.2 %). There was no significant statistical difference in the occupation distribution of respondents considering both periods, in almost all hospitals except for hospital 2 (Table 1).

In relation to safety perception in the OR (Table 2), there was an improvement of favorable responses from nursing staff and anesthesiologists (p = 0.001 and p = 0.046, respectively) after checklist intervention. Regarding the need for verifications before surgery, only the nursing staff showed a significant statistical increase in the post-intervention period (p < 0.001). As for the anesthesiologist and surgeon teams, there was no significant change after intervention. The assessment regarding patient safety concerns in the OR improved significantly among the nursing team (p < 0.001). Regarding the agreement on teamwork implementation (question 5), there was a significant increase in the proportion of favorable responses in the post-intervention period among nursing staff and anesthesiologists (p < 0.001 and p = 0.038, respectively). In relation to the frequency professionals ignore rules or surgical safety standards and hand hygiene (items 6–8), there was a high percentage of those who never ignored the post-intervention rules among the nursing team, which is a statistically significant response (p < 0.001).

**Table 2:** Surgical team safety attitudes, surgical safety checklist according to intervention period (period I—pre and period II—post)

Questions^a^	Response		Total		Nursing team		Anesthesia team		Surgeon team	
			Period I	Period II	Period I	Period II	Period I	Period II	Period I	Period II
1	Yes									
	No. (%)		193 (72.8 %)	189 (84.4 %)	80 (73.4 %)	100 (90.1 %)	32 (62.7 %)	32 (82.1 %)	58 (74.4 %)	46 (79.3 %)
	p value**		0.002		0.001		0.046		0.502	
2	Essential									
	No. (%)		195 (72.0 %)	190 (81.9 %)	76 (67.9 %)	103 (89.6 %)	46 (90.2 %)	34 (87.2 %)	54 (67.5 %)	41 (67.2 %)
	p value**		0.009		<0.001		0.654		0.971	
3	Yes									
	No. (%)		187 (70.3 %)	186 (83.8 %)	72 (64.9 %)	103 (93.6 %)	37 (74.0 %)	30 (78.9 %)	61 (77.2 %)	46 (80.7 %)
	p value**		<0.001		<0.001		0.592		0.626	
4	Very easy									
	No. (%)		32 (12.1 %)	60 (26.3 %)	13 (12.1 %)	35 (32.7 %)	10 (20.0 %)	9 (25.7 %)	7 (8.8 %)	15 (32.6 %)
	p value**		<0.001		0.001		0.679		0.009	
5	Yes									
	No. (%)		175 (66.3 %)	191 (83.0 %)	74 (67.9 %)	101 (88.6 %)	28 (54.9 %)	29 (76.3 %)	54 (70.1 %)	46 (75.4 %)
	p value**		<0.001		<0.001		0.038		0.492	
6	Never									
	No. (%)		14 (5.2 %)	116 (50.0 %)	8 (7.2 %)	76 (66.1 %)	3 (5.9 %)	17 (43.6 %)	2 (2.5 %)	22 (36.1 %)
	p value**		<0.001		<0.001		<0.001		<0.001	
7	Never									
	No. (%)		5 (1.9 %)	118 (52.0 %)	1 (0.9 %)	78 (69.6 %)	2 (3.9 %)	17 (44.7 %)	1 (1.3 %)	22 (36.7 %)
	p value**		<0.001		<0.001		<0.001		<0.001	
8	Never									
	No. (%)		11 (4.1 %)	119 (52.7 %)	5 (4.5 %)	79 (70.5 %)	4 (8.2 %)	17 (47.2 %)	1 (1.3 %)	22 (36.1 %)
	p value**		<0.001		<0.001		<0.001		<0.001	

^a^ *1* If you were treated as a patient in this hospital, would you feel safe?; *2* How do you assess the need to perform verifications or checks in the OR before a surgical procedure?; *3* Have your colleagues ever encouraged you to report any concern you may have had in relation to patient safety?; *4* How does communication with the team in the OR take place before any issue during patient care?; *5* Do you think health professionals work well in groups, as a well-integrated team?; *6* How often do health professionals ignore surgical safety rules or standards established for the OR: Hand hygiene?; *7* How often do health professionals ignore surgical safety rules or standards established for the OR: Regulations and routines?; *8* How often do health professionals ignore surgical safety rules or standards established for the OR: surgical technique?

** p value calculated based on the proportion test for each distribution difference between periods I and II

The majority of respondents thought that the checklist was easy to use. However, a significant difference was noted in the proportions of professionals (p = 0.008). The checklist was considered by most respondents quick to use, with a significant difference (p = 0.012). The question of the use of the checklist in case the respondents were to undergo surgery themselves showed no significant difference in the proportional distribution in relation to the groups (Table 3). Most groups believed that checklist implementation improved communication: 92.7 % of the nursing team, 87.9 % of the anesthesiologists and 75.6 % of the surgeons, with significant proportional differences (p < 0.001). At least 90.0 % of respondents from each team agree that the checklist helps reduce OR errors, with statistical difference between the teams. Moreover, although most respondents also agree that the checklist helps to develop surgical safety culture in the unit, there are significant differences among teams, with 96.4 % in the nursing team, 93.9 % of anesthesiologists and 78.7 % of surgeons (Table 3).

**Table 3:** Surgical team opinions towards the WHO surgical safety checklist implementation in hospitals in the Brazilian Federal District

Questions	Total		Nursing team		Anesthesia team		Surgeon team		p value^a^
	No.	%	No.	%	No.	%	No.	%	
1^b^									
Hard to use/fill out	12	5.5	5	4.5	0	0.0	7	13.0	0.008
Easy to use/fill out	207	94.5	106	95.5	39	100.0	47	87.0	
Total	219	100.0	111	100.0	39	100.0	54	100.0	
2^b^									
Brief and quick	206	92.0	105	92.9	39	100.0	49	86.0	0.012
Extensive and time consuming	18	8.0	8	7.1	0	0.0	8	14.0	
Total	224	100.0	113	100.0	39	100.0	57	100.0	
3^b^									
Yes	228	98.3	113	98.3	39	100.0	59	96.7	0.739
No	4	1.7	2	1.7	0	0.0	2	3.3	
Total	232	100.0	115	100.0	39	100.0	61	100.0	
4^b^									
Yes	172	87.8	102	92.7	29	87.9	31	75.6	<0.001
No	24	12.2	8	7.3	4	12.1	10	24.4	
Total	196	100.0	110	100.0	33	100.0	41	100.0	
5^b^									
Yes	217	96.9	113	99.1	39	100.0	51	89.5	<0.001
No	7	3.1	1	0.9	0	0.0	6	10.5	
Total	224	100.0	114	100.0	39	100.0	57	100.0	
6^b^									
Yes	185	91.1	106	96.4	31	93.9	37	78.7	<0.001
No	18	8.9	4	3.6	2	6.1	10	21.3	
Total	203	100.0	110	100.0	33	100.0	47	100.0	

^a^p value calculated based on the proportion test for each distribution difference between Periods I and II

^b^ *1* In relation to the checklist usability would you consider it: ^b^ *2* In relation to checklist completion, do you consider it to be a tool? ^b^ *3* If you were submitted to surgery, would you like the checklist to be used? ^b^ *4* Was communication improved by using the checklist? ^b^ *5* Does the checklist help avoid errors in the OR? ^b^ *6* Has the checklist contributed to developing a surgery safety culture in the OR

## Discussion

The use of the WHO surgical safety checklist has been mandatory in several countries [15, 18, 19], and in Brazil, national laws and health regulations [20–24] and guidelines [25, 26] were published for the safety of surgical care in health services. The implementation of the checklist in these health units implies an integrated team effort, with greater participation and surgical team situation awareness. In addition, several studies show that the WHO surgical safety checklist can modify personal attitudes of professionals working in ORs [10, 27, 28] and is seen as a tool that improves patient safety [29].

In this study, the members of the participating surgical teams (surgeons, anesthesiologists and nursing staff) had an average of 12 years of experience in the healthcare area and only 6 years in the current surgical unit. The relative amount of team experience indicates a need to encourage the implementation of patient safety actions. This will be reflected in the work process in the operating room, with improvement of the attitudes and opinions regarding the applicability of the checklist, which could increase the empowerment of the team as a whole, which, in turn, could be expressed as a decrease in the occurrence of potential harm to the surgical patient.

The survey revealed that, regarding checklist application, most of the nursing staff (92.9 %) and 100.0 % of anesthesiologists indicated that the checklist is a brief and quick tool, while, in contrast, 14.0 % of surgeons considered it as extensive and time consuming. Recent research suggests that nurses have more positive attitudes towards filling out the checklist compared to surgeons [4, 30, 31]. It is important to recall that the nursing profession is predominantly occupied by females in Brazil and therefore this result could have been influenced by gender. It is noteworthy to mention that some health professionals may have skeptical attitudes towards checklist implementation and/or in relation to changes in their routines since they associate it with a significant expansion in their workloads. However, the checklist only formalizes the tasks to be performed without adding tasks to services [32]. Besides, the filling time is singular and takes from 2 to 3 min [33].

Regarding communication between surgical team members, according to most respondents (87.8 %), with an emphasis on the nursing teams (92.7 %) and anesthesiologists (87.9 %), the use of the checklist improved communication. Similar results were reported in other studies [34]; i.e., checklist use helped improve communication within the team [34–37] or between multi-professional teams [38]. It is important to mention that miscommunication between team members is a common cause of AEs or sentinel events (never events), such as surgeries performed on the wrong body part. The US Joint Commission reports showed that out of 843 EAs registered, almost 70.0 % of the cases were related to communication problems [38]. In Brazil, about 8000 incidents were reported by patient safety centers (NSP) to the National Health Surveillance System (SNVS) in 2014, which included 216 never events: five cases of retained foreign objects in a patient after a surgical procedure and one related to intraoperative death or immediate postoperative death in an ASA Class 1 patient [39]. A study with 3231 orthopedic surgeons during the 44th Brazilian Congress of Orthopedics and Traumatology, in 2012, designed to investigate awareness and knowledge about the WHO surgical safety checklist among Brazilian orthopedic surgeons, showed that 40.8 % of the 502 respondents reported having experienced wrong-site or wrong-patient surgery and 25.6 % of them reported “miscommunication” as a cause for the error [40].

In this study, when asked if the checklist would help prevent errors in the OR, 217 surgical team members (96.9 %) responded affirmatively, highlighting the nursing teams (99.1 %) and anesthesiologists (100.0 %). Similar to the studies conducted by Haynes et al. [6], about 80.0 % of the respondents stated that the checklist prevented the occurrence of errors. In other words, surgical team awareness about the potential of this tool to prevent damage to patients in the OR environment is evident. In a descriptive study carried out in three hospitals in Guatemala in 2011 [10], 1 year after local implementation of the checklist, when professionals were asked if errors committed in the OR could have been avoided by making use of the checklist, 42.5 % (17) of the resident surgery physicians, 63.2 % (28) of the resident anesthesiologists, 100.0 % (6) of the anesthesiologists and 35.7 % (20) of the nursing team gave an affirmative answer. In turn, most of the errors that take place during surgeries can also be attributed to failures related to non-technical skills that the checklist aims to improve, such as situational awareness, the decision-making process, communication between the surgical team and team leader during the performance of tasks [41].

Most respondents in this study (91.1 %) calling attention to the nursing teams (106) and anesthesiologists (31), stated that the checklist has helped to develop a surgical safety culture in the OR, which may suggest sustained advances in the current regulations in force in Brazil, confirming changing attitudes towards safety in health services. It should be noted that ANVISA RDC Resolution No. 36/2013 [20] defines safety culture as “a set of values, attitudes, skills and behaviors that determine the commitment to health and safety management, replacing guilt and punishment with the opportunity to learn from failures and improve health care”. In a cross-sectional study conducted during the 97th Annual Congress of the Swiss Society of Surgery in 2010, where the opinions of 138 surgeons and anesthesiologists towards the checklist were surveyed, the majority of respondents (75.4 %) agreed that the checklist helps develop a safety culture in the OR (34), which is also corroborated by the WHO [5]. Research reports that briefing sessions supported by a checklist help maintain an open culture of professional development in the workplace, where training is improved through the exchange of information, communication and opportunity to express one’s opinion [42]; i.e., it serves as an anchor for surgical safety culture in the OR.

In this study, it was also found that 98.3 % of participants expressed that they would like the checklist to be used should they themselves be subject to surgery, suggesting that the tool had good acceptance by most participant surgical teams. This finding was similar to the one reported by Haynes et al. [6] in their study, in which 93.0 % of the participants responded positively to that question. In 2015, a study to assess the perceptions of 1852 members of surgical teams towards several dimensions of surgical safety with the use of a checklist (35 items) in 38 hospitals in South Carolina (USA), showed that almost all members of the teams (1237/1852) indicated a preference for using this tool if they themselves would be submitted to a surgical procedure [43]. It can be concluded, thus, that the checklist reinforces organizational safety culture and improves communication among the surgical team, and that it is not about mere operational formality or the implementation of a superficial instrument. It replaces, indeed, the illusion of safety with a guiding tool for positive results for the patient in the OR [43].

Regarding participants’ attitudes (sense of safety in relation to the hospital), although a significant improvement of favorable response in period II can be noted among all professionals, including nursing teams and anesthesiologists, such an improvement was not observed among surgeons. Singer et al. [43] found that 15.0 % of all surgical team members and up to 57.0 % in other hospitals indicated that they would not feel safe as a patient in their own ORs. According to the authors, the successful implementation of the checklist should lead to improved surgical safety culture and attitudes in the OR and, as a consequence, lead practitioners to experience greater perceived safety in the care provided. Hence, surgical team members could feel safe as patients in the units where they work [44], similar to the results found in this study.

The need for checks before the surgical procedure and patient safety concerns showed significant improvement only among the nursing team (questions 2 and 3, respectively). Studies with similar responses to this sample, as in Haynes et al. [6], found strong agreement among surgical team members in the post-intervention period in relation to checklist implementation, considering the same variables in hospitals in the Brazilian Federal District.

Limitations of this study are: the number of participating hospitals, the specific populations in each health service who responded to the instrument, the characteristics of surgical teams, a variety of hospital environments, as well as the several different cases admitted to the OR during the study. Thus, the results may not be applicable to all units. On the one hand, there is the possibility of biases inherent to the prospective design (pre- and post-intervention), because members of surgical teams were aware that the checklist was being implemented and assessed, which may have influenced the provision of answers demonstrating the success of its implementation. On the other hand, the implementation of the checklist itself, together with the training conducted in the hospitals surveyed may have improved communication and therefore led to a better understanding of teamwork and safety culture in these services. Additionally, various laws on patient safety were established in Brazil during the research, including the establishment of the National Program of Patient Safety (PNSP) [21] and ANVISA’s regulation on the issue [20], as well as the protocol for safe surgery [24], which may have led to better assimilation and or understanding of the importance of surgical safety by the participating teams.

## Conclusions

In general, the study results indicate progress in the attitudes and opinions of surgical safety teams towards the checklist in the assessed units, contributing to further improvement of safety in the OR. However, given the difficulties found in the implementation of the checklist, especially as it relates to the acceptance of the referred instrument by surgeons, it is urgent that health planners draw up new strategies to deal with the problem, in an attempt to make surgeons understand that this is a work procedure, without a hierarchy in which some professionals are dominant over others, with the empowerment of all team members and where care is centered on the patient. That is, it is essential that teams override purely technological aspects and make advances in interpersonal relations and communication, with the participation and responsibility of all members in order to increase surgical patient safety.

Furthermore, research on safety culture measurement is suggested, including qualitative studies on the subject in order to clarify these difficulties, as well as cost-effectiveness analyses of the checklist in surgical centers in the country. The assessment of patient safety perceptions and behaviors of members of surgical teams allows one to identify the most vulnerable areas so that managers and leaders can promptly intervene in order to solve problems in the OR. Ultimately, methodological strategies for motivational training grounded in appropriate communication with a transversal axis can institute team empowerment, with a focus on a work process that is centered on the patient and that favors attitudes regarding safety. Finally, new studies are necessary in order to verify the sustainability of the surgical teams’ changes in attitudes towards safety in the hospitals studied.

## Abbreviations

AEs: adverse events
ANVISA: National Health Surveillance Agency, Brazil
DF: Federal District
IQR: interquartile deviation
NSP: patient safety centers
OR: operating room
PNSP: National Program of Patient Safety
SAQ: safety attitudes questionnaire
SNVS: National Health Surveillance System
SSI: surgical site infection
UnB: University of Brasilia
WHO: World Health Organization
